# A model to quantify the influence of treatment patterns and optimize outcomes in nAMD

**DOI:** 10.1038/s41598-022-06362-w

**Published:** 2022-02-18

**Authors:** Focke Ziemssen, Hansjürgen Agostini, Nicolas Feltgen, Robert P. Finger, Christos Haritoglou, Hans Hoerauf, Matthias Iwersen, Martina Porstner, Andreas Clemens, Benjamin Gmeiner

**Affiliations:** 1 Department of Ophthalmology, University Hospital Leipzig, Leipzig, Germany; 2 Center for Ophthalmology, University Eye Hospital Tübingen, Tübingen, Germany; 3 Eye Center, Medical Center, Faculty of Medicine, University of Freiburg, Freiburg, Germany; 4 Department of Ophthalmology, University Medical Center Goettingen, Goettingen, Germany; 5 Department of Ophthalmology, University of Bonn, Bonn, Germany; 6 Herzog Carl Theodor Eye Hospital, Munich, Germany; 7 Novartis Pharma GmbH, Roonstrasse 25, 90429 Nürnberg, Germany; 8 Novartis Pharma AG, Basel, Switzerland; 9 Department of Cardiology and Angiology I, Heart Center Freiburg University, Faculty of Medicine, University of Freiburg, Freiburg, Germany; 10 Department of Chemistry and Chemical Biology, Harvard University, Cambridge, MA USA

**Keywords:** Health care, Diseases, Eye diseases, Macular degeneration, Medical research, Outcomes research

## Abstract

Neovascular age-related macular degeneration (nAMD) is a progressive retinal disease that often leads to severe and permanent vision loss. Early initiation of anti-vascular endothelial growth factor (anti-VEGF) therapy has been shown to preserve vision in nAMD patients. Concurrently, treatment outcomes in real-world are inferior to those reported in clinical trials. The most likely reasons observed are fewer treatment-intensity in routine clinical practice than in clinical trials. The other possibility could be the delay in starting treatment and the re-treatment interval. Although a negative impact of aforementioned parameters seems obvious, quantitative impact measures remain elusive in a real-world setting due to a lack of an ‘optimal treatment’ control group. To overcome this shortcoming, we developed, validated, and applied a model to assess and quantify the impact of anti-VEGF administration variables on visual acuity development in a prospective nAMD patient cohort. The model was further applied to probe the impact of the COVID-19 pandemic on visual progressions in nAMD patients. The presented model paves the way to systematically explore and evaluate realistic interventions in the current treatment paradigm, that can be adopted in routine clinical care.

## Introduction

Neovascular age-related macular degeneration (nAMD) is a progressive retinal disease that leads to severe and permanent visual impairment and legal blindness if not treated. Early initiation of anti-vascular endothelial growth factor (anti-VEGF) therapy has shown to preserve vision of patients with this disease^1–4^. Current treatment schemes usually include 3 injections at monthly interval (q4w) followed by an individualized treatment regimen like *Pro Re Nata (PRN)*, *Treat and Extend (T*&*E),* or *Observe and Plan* with regular optical coherence tomography (OCT) controls to detect early reactivation of the disease^5–7^. Anecdotally, some physicians do not start treatment with three loading injections but treat and extend immediately from start of treatment.

Randomized controlled trials (RCT) showed that individualized PRN and T&E are not inferior to fixed treatment regimens but mitigate treatment burden for the patient^8–10^. However, the average number of intravitreal injections applied by PRN or T&E in real-world setting is consistently lower than in RCTs due to multifactorial reasons including the patient, physician, treatment center set up and logistical factors^11–15^. Concurrently, VA gains observed in real-world settings stay below those observed in RCT. While about eight injections where applied on average in the first year of PRN and T&E arms within RCTs^8–10^, means of 4–7 injections in the first year were observed in real-world studies^11–13^.

Furthermore, treatment start is commonly delayed after the first detection of disease signs. Reasons include a lack of awareness of the disease and its urgency by the patient or primary care giver and logistical constraints at the patient, care giver or physician/center^16^. During the delay, the disease progresses and may cause irreversible damage to the retina. There is increasing evidence that immediate treatment without delay after diagnosis improves and any delay of more than two to three weeks worsens treatment outcomes^16,17^. Beside the challenges we see in routine clinical practice, the COVID-19 pandemic exemplified the impact of may have aggravated the situation of undertreatment, since as investigations examinations or injections were omitted or delayed^18–20^.

To better understand impact of number of injections, treatment delay and other baseline characteristics on treatment outcomes, a model was developed to assess and quantify their impact on mean VA progression in a prospective patient cohort. Therefore, the dataset of a non-interventional trial cohort was used to inform such a general model structure and to validate it using a second dataset. Similar models were successfully used in RCT data^21–23^, but have not been adapted to real-world data from non-interventional studies (NIS) yet. As real-world data are very relevant for day-to-day clinical decisions, our study is needed to close this gap.

## Methods

### Study cohorts and data

The present modelling study uses datasets from 3 studies: 2NIS and one RCT. The dataset from the NIS OCEAN^24^ (NCT02194803) was used for model training and scenario development. Datasets from the NIS PACIFIC (NCT04847895) and the RCT HARBOR^9^ (NCT00891735) were used as independent validation datasets. Table 1 provides a description of the study designs and cohorts.

**Table 1:** Summary of studies used for model training (NIS OCEAN) and validation (NIS PACIFIC, RCT HARBOR).

	NIS OCEAN *Training dataset*	NIS PACIFIC *Validation dataset 1*	RCT HARBOR^1^ *Validation dataset 2*
**Study**			
Time of data collection	2011–2016	2015–2021	2009–2011
Design	NIS	NIS	RCT
Duration per patient	2 years	2 years	1 year
**Number of nAMD patients in study at**			
Baseline	3631	2973	275
6 months	2943	2069	n/a
12 months	2424	1602	258
18 months	1928	1251	n/a
**Treatment**			
Lucentis treatment regimen	Acc. to label	Acc. to label	q4w
Lucentis dose (mg)	0.5	0.5	0.5
Number of injections per year, mean (SD)	4.47 (2.21)^2^	4.97 (2.87)^2^	11.3 (1.8)^2^
Treatment delay (days), mean (SD)	20.3 (19.4)	9.7 (14.6)	8.3 (n/a)^3^
**Patients**			
Male, %	38.6	40.2	41
Age (years), mean (SD)	77.8 (8.2)	79.0 (8.2)	78.8 (8.4)
Country	Germany	Germany	United States
Baseline VA, mean (SD)	52.1 (21.3)	55.1 (22.1)	54.2 (13.3)
**Pretreatment status, %**			
Treatment-naïve	73.2	44.1	100
Pretreated with anti-VEGF	17.4	55.9	0
Possibly pretreated	9.5	n/a	0

*nAMD* neovascular age-related macular degeneration; *NIS* non-interventional study; *RCT* randomized controlled trial; *SD* standard deviation; *VA* visual acuity; *VEGF* Vascular Endothelial Growth Factor.

^1^Only HARBOR patients of the ranibizumab 0.5 mg q4w arm if not indicated otherwise.

^2^1st year.

^3^All HARBOR patients (n = 1097).

All patients provided written informed consent before screening or initiation of any study-related procedures. The original OCEAN study protocol was approved by the ethics committee of the Eberhard-Karls-University, Tübingen, Germany. The original PACIFIC study protocol was approved by the ethics committee of the Bavarian State Board of Physicians (Bayerische Landesärztekammer), Germany. The original HARBOR study protocol was approved by independent institutional review boards at all 99 study sites prior to the start of the study^9,25^. All trials were conducted in accordance with principles of the Declaration of Helsinki, International Conference on Harmonization E6 Good Clinical Practice Consolidated Guideline, and other regulations as applicable and were compliant with the Health Insurance Portability and Accountability Act of 1996.

### Model development

**Figure 1:**
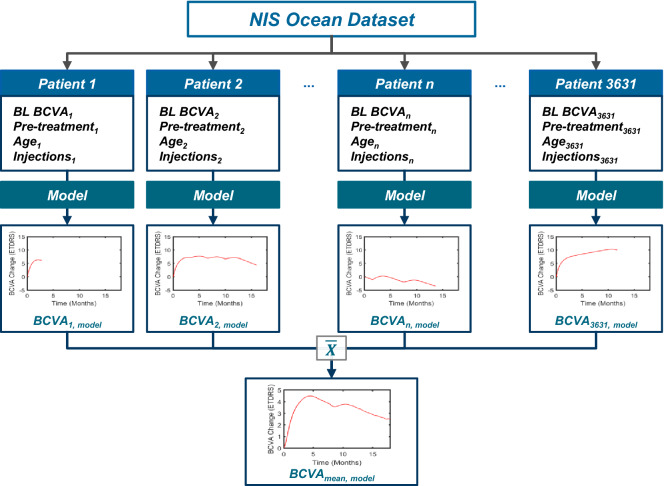
Development of visual acuity prediction model based on individual baseline characteristics (BL BCVA, age and pre-treatment status) and the time and dose of anti-VEGF intravitreal injections. The model was trained using real-world data from the non-interventional study OCEAN. BCVA = best corrected visual acuity; BL = baseline; VEGF = Vascular Endothelial Growth Factor.

The model is an indirect response pharmacokinetic (PK)/pharmacodynamic (PD) drug-disease model that simulates individual VA progression of patients with nAMD who are treated with ranibizumab. VA is simulated dependent on patient's baseline data (age, baseline VA, pretreatment status) and the dose and timing of intravitreal ranibizumab injections (Fig. 1). Focus of the model is on prediction of population level mean VA progression, not on individual prediction.

The general model structure was first developed by Holz et al.^21^ based on data of the RCTs MARINA, ANCHOR, and PIER. It was then extended and applied by Mulyukov et al.^22^ and Agostini et al^23^*.* In the present study, the model is extended and applied for the first time to real-world data. Model training was on the dataset from the NIS OCEAN, validation was on the independent dataset from the NIS PACIFIC and the RCT HARBOR. The present model structure was adopted from Mulyukov et al. and expanded further to a pre-treated patient population.

Natural progression of the disease without treatment is modelled by the differential equation

1 $$\frac{{dg}_{i}\left(t\right)}{dt}={k}_{in}-{k}_{out}\cdot {g}_{i}\left(t\right)$$

2 $${g}_{ss}=\frac{{k}_{in}}{{k}_{out}}$$

3 $${g}_{i}\left(0\right)={g}_{0,i}$$

where ${g}_{i}\left(t\right)$ is the VA of subject *i* at time *t*, ${g}_{ss}$ is the stationary value the expected VA approaches, ${k}_{out}$ is the VA deterioration rate due to the disease, ${k}_{in}$ is a rate parameter given by ${g}_{ss}$ and ${k}_{out}$, and ${g}_{0,i}$ is the baseline BCVA of subject *i*.

The effect of intravitreal anti-VEGF injections is modelled as effect on ${k}_{in}$:

4 $$\frac{{dg}_{i}\left(t\right)}{dt}={k}_{in}\cdot \left(1+{E}_{max, i}\left(t,{\alpha }_{i}\right)\cdot \frac{{C}_{i}\left(t\right)}{{EC}_{50}+{C}_{i}\left(t\right)}\right)-{k}_{out}\cdot {g}_{i}\left(t\right)$$

where ${C}_{i}\left(t\right)$ is the drug concentration in the vitreous of subject *i* at time *t*, ${E}_{max,i}\left(t,{\alpha }_{i}\right)$ is the maximum possible treatment effect of the drug modelled as a function of pre-treatment status and time and ${EC}_{50}$ is the concentration when half of the maximum effect is reached.

${C}_{i}\left(t\right)$ is modelled by first-order elimination pharmacokinetics with fixed elimination rate constant ${k}_{drug}$ known from literature data. ${E}_{max,i}\left(t,{\alpha }_{i}\right)$ is assumed to decrease with time after baseline according to:

5 $${E}_{max,i}\left(t,{\alpha }_{i}\right)={E}_{max,i}^{ss}+{\alpha }_{i}\cdot \Delta {E}_{max}^{0}\cdot {e}^{-{k}_{{E}_{max}}\cdot t}$$

to consider a larger effect of the first loading injections, where ${E}_{max,i}^{ss}$ is the steady state of ${E}_{max}$, ${E}_{max,i}^{ss}+{{\alpha }_{i}\cdot \Delta E}_{max}^{0}$ is ${E}_{max}$ at baseline, ${\alpha }_{i}$ is a scaling parameter for pre-treatment status and ${k}_{{E}_{max}}$ is the rate at which ${E}_{max}$ decreases.

${E}_{max,i}^{ss}$ is dependent on the patient's age on log-scale:

6 $$\mathit{log}\left({E}_{max,i}^{ss}\right)=\mathit{log}\left({E}_{max,0}^{ss}\right)+\beta \cdot \mathit{log}\left(\frac{{age}_{i}}{77}\right)$$

The model was parametrized by using least square estimation on data of the NIS OCEAN. Further details on the model can be found in the supplementary information.

### Model validation

The parameterized model was used to predict the observed mean VA progression from baseline in the independent validation datasets of the NIS PACIFIC and the RCT HARBOR, based on observed baseline parameters (baseline VA, age and pre-treatment status) and timing of ranibizumab injections. For PACIFIC, individual scale data were used. Each study patient was individually simulated based on its respective patient individual, baseline and injection data until the time of drop out or completion of the study. Patients with missing baseline data were not considered. For HARBOR, age and baseline VA were drawn from normal distributions with mean and standard deviation from the demographics table of the study (see Table 1). According to the study design, no pre-treatment was assumed. Ranibizumab injections were simulated as 3 q4w loading injections starting at baseline followed by a q8w scheme at a single dose of 0.5 mg until Month 12. Mean BCVA gain and 95% prediction intervals for the mean were calculated based on the simulated patient data at each time point.

### Application on real-world scenarios within the model

The validated model was used to predict mean VA gain under modified treatment scenarios based on real-world data of the OCEAN study and under a scenario simulating the impact of COVID-19 pandemic. In all scenarios based on OCEAN, the number of patients, individual baseline parameters and the observation time per patient were kept as in the OCEAN study. In the scenarios on delayed treatment start and undertreatment/loading period, also the total number of injections per patient was kept as in the OCEAN study.

#### Interpretation of the OCEAN results compared with 3l-q8w (8 weekly) treatment or the natural cause of nAMD (no treatment)

The real-world injection pattern of the OCEAN study was first compared to two extreme scenarios: (i) A 3l-q8w pattern. Start of the treatment without delay, with 3 loading injections in q4w, followed by maintenance injections with q8w. The first-year injection frequency in this pattern corresponds to the mean injection frequency observed in PRN arms in RCT^8–10^. (ii) No treatment (natural progress of the disease).

#### Consequences of delayed treatment start

Consequences of delayed treatment start were explored in a scenario where the delay was reduced to zero for all patients and a scenario where the delay was bound at a maximum of 2 weeks. In both scenarios, the number and timing of injections after the first injection was unchanged compared to the OCEAN study (i.e., individual injections were shifted forward and the gap without injections was shifted from the beginning to the end of the observation time).

#### Effect of undertreatment and loading period

The effect of an initial loading period with 3 injections q4w was explored in 2 scenarios where treatment delay and the total number of injections per patient until Month 12 were kept as in the OCEAN study, but the timing of injections was modified in 2 ways: (i) scenario with loading period (treatment started with 3 loading injections q4w; the remaining injections were distributed equally spaced over the remaining observation time until Month 18; the exact time point of injections was varied by drawing from normal distribution with σ = 4 for loading injections and σ = 7 for maintenance injections); (ii) scenario without loading period (after the first injection, all remaining injections were distributed equally spaced over the remaining observation time until Month 18). In an additional scenario, the real-world treatment pattern was substituted by a 3l-q8w pattern (3 loading injections q4w followed by maintenance injections q8w) as reference of a pattern without undertreatment.

#### Impact of COVID-19

We simulated a realistic scenario of missed intravitreal injections similar to the situation during the COVID-19 pandemic and its consequences for VA outcome. The simulated population consisted of 450,000 nAMD patients, corresponding roughly to the German nAMD population^26,27^. The baseline characteristics age and baseline VA were randomly drawn from the OCEAN study (Table 1). Baseline was randomly assigned between 01.09.2019 and 01.03.2021 and all patients were simulated from their individual baseline until 01.03.2021. All patients were not pre-treated at baseline. Regular (i.e., without COVID-19 impact) individual injection patterns were randomly drawn from data of the OCEAN study, including the delay between baseline and treatment start.

The impact of missed injections due to COVID-19 pandemic was simulated by assigning each injection of the regular pattern a probability that the injection was actually performed. The probability was calculated from the estimated total number of ranibizumab injections performed in Germany in a specific week ${N}_{inj}\left(t\right)$ relative to the distribution numbers before the COVID-19 pandemic $\overline{{N }_{inj}}$:

7 $$P\left(inj|t\right)=\mathrm{min}\left(\frac{{N}_{inj}\left(t\right)}{\overline{{N }_{inj}}},1\right)$$

The number of ranibizumab injections per date in Germany from 01.01.2020 to 01.03.2021 was estimated from daily distribution numbers of Lucentis in Germany.

## Results

### Parameterized model

A total of 3,631 patients with 65,673 VA observations from baseline until Month 24 informed the model. Parameter estimates are listed in Supplementary Table S1.

### Model validation

**Figure 2:**
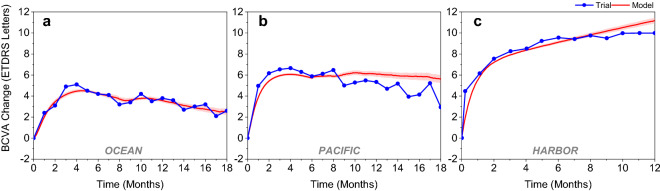
Mean BCVA change from baseline: model prediction with 95% prediction interval (red) versus observed data (blue) for (**a**) the trainings dataset OCEAN, (**b**) the non-interventional study validation dataset PACIFIC, and (**c**) the randomized controlled trial validation dataset HARBOR. BCVA = best corrected visual acuity.

The model accurately predicted mean VA gains in the OCEAN study that was used for training and in the independent validation datasets of the NIS PACIFIC and the RCT HARBOR (Fig. 2). In the prediction of independent data, only slight underprediction occurred in the first 8 months and slight overprediction in later months. As a measure of goodness of fit between model prediction and observed data, adjusted R^2^ was calculated for the mean VA gain over time. It was high for all 3 studies, OCEAN (training data): adjusted R^2^ = 0.91; PACIFIC (independent validation): adjusted R^2^ = 0.81; HARBOR (independent validation): adjusted R^2^ = 0.94.

Goodness of fit was also high for patient subgroups with specific injection patterns. In subgroups of the OCEAN study with small (< 14 days, n = 1587) and large (> 28 days, n = 1081) delay between baseline and treatment start, adjusted R^2^ was 0.90 and 0.87, respectively. Smaller R^2^ in these subgroups than in the total population was also due to the smaller sample size.

Known effects like the effect of age and the ceiling effect of baseline BCVA on BCVA gain were predicted by the model (see Supplementary Figures S2, S3).

The model was designed to predict mean BCVA gain at population level; it is not possible to predict individual BCVA gain using the model. Supplementary Figure S1 shows how adjusted R^2^ increases with increasing population size. At n = 1, which corresponds to individual prediction, and below n = 50, adjusted R^2^ is only about 0.2. At sample sizes ≥ 500, adjusted R^2^ is ≥ 0.8. Apparently, there is a large proportion of individual variation that is not explained by the modelled characteristics age, baseline BCVA, pre-treatment status and timing and dose of injection, whereas on population level, the mean outcome is well predicted by these features. For the simulation in Supplementary Fig. S1, samples of a specific number of patients were drawn without replacement from the OCEAN population and adjusted R^2^ was calculated for the sample. Mean and 95% prediction intervals for adjusted R^2^ were calculated from 30 draws per sample size.

### Results of real-world scenarios

#### Interpretation of the OCEAN results compared with 3l-q8w (8 weekly) treatment or the natural cause of nAMD (no treatment)

**Figure 3:**
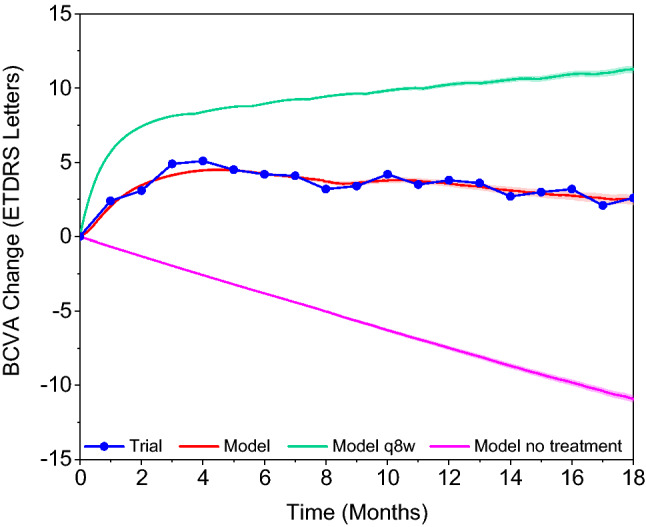
Model prediction for mean BCVA change from baseline with 95% prediction interval in a 3l-q8w treatment scenario (green) and a scenario without treatment (purple) vs. the real-world scenario of OCEAN (blue, red). The 3l-q8w scenario consists of 3 loading injections q4w without delay between baseline visit and first injection, followed by injections q8w until Month 18. Baseline characteristics are as in OCEAN. BCVA = best corrected visual acuity.

Mean VA gain in the real-world OCEAN scenario was estimated to be between the hypothetical scenario with 3l-q8w injections applied without delay and the no-treatment scenario (Fig. 3). The difference between the 3l-q8w and the OCEAN scenario depicts the overall VA loss due to real-world treatment as it occurred in the OCEAN study. In OCEAN, the first VA increase was not as steep and not as high as in the best-case scenario. After about 4.5 months, mean VA slowly decreased in OCEAN while it continued increasing in the best-case treatment scenario before the onset of saturation.

#### Consequences of delayed treatment

**Figure 4:**
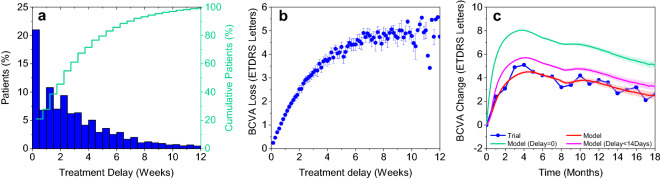
Consequences of delay between diagnosis and first injection on visual acuity. (**a**) Observed treatment delay in OCEAN. (**b**) BCVA loss due to treatment delay in Month 18 (mean ± 95% prediction interval). (**c**) Mean BCVA change from baseline with 95% prediction interval: model prediction for a scenario with no treatment delay (green) and delay limited to a maximum of 2 weeks (purple) but frequency of injections and baseline characteristics as in OCEAN vs. the real-world scenario of OCEAN (blue, red). BCVA = best corrected visual acuity.

In the OCEAN study, there was a substantial delay between the first diagnosis of active nAMD and the treatment start for most patients (Fig. 4a). The mean delay was 20.3 ± 19.4 days (Table 1). 43.7% of patients were treated earlier than 2 weeks after first nAMD diagnosis; for 29.1% of patients, the delay was more than 4 weeks.

Figure 4b displays the loss of BCVA in Month 18 (or the last value in case of early discontinuation) as a function of treatment delay. BCVA loss increased steeply until a delay of about 4 weeks and less steeply until about 8 weeks delay. Even a delay of about 2 weeks resulted in a mean loss of about 2.5 letters. A delay of about 4 weeks results in a mean loss of about 4 letters.

Reducing the delay to a theoretical minimum of zero had a significant effect on VA outcome (Fig. 4c, green line). Mean BCVA increase in the first 4 months of treatment was significantly steeper than in the real-world scenario. Thereafter, mean BCVA decreased in both scenarios, but the difference in VA between the scenarios was maintained through Month 18. Reducing the delay to a maximum of 2 weeks had a similar but less pronounced effect (Fig. 4c, purple line).

#### Effect of undertreatment and loading period

**Figure 5:**
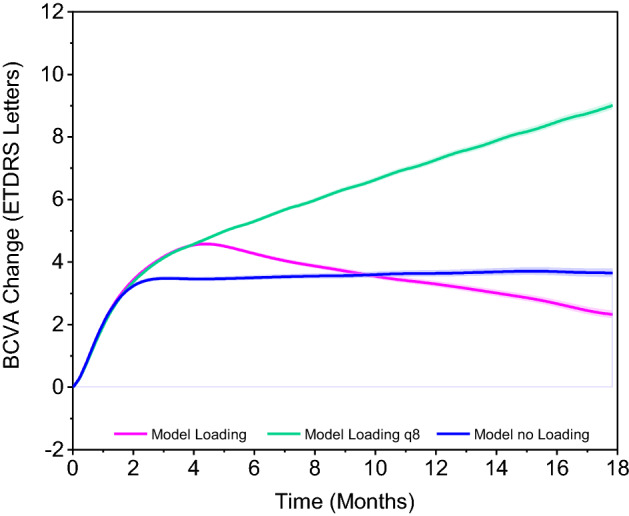
Effect of undertreatment and loading period. Mean BCVA change from baseline with 95% prediction interval in a scenario with 3l-q8w pattern (green), a scenario with 3 q4w loading injections followed by equally spaced maintenance injections (purple) and a scenario without loading injections with injections equally spaced throughout the observation time (blue). In the purple and blue scenario, the total number of injections, treatment delay and baseline characteristics are as in OCEAN in both scenarios. BCVA = best corrected visual acuity.

VA gain in the scenarios based on the real-world injection frequency stayed after 5 months significantly below the scenario with 3l-q8w (Fig. 5). The difference between the two scenarios based on real-world injection frequency with and without loading period was moderate. Maximum VA gain in the first 5 months was about 1 letter higher in the scenario with loading period than without loading period. In the long term, VA loss after the initial peak was greater in the scenario with loading period, leading to superiority of the scenario without loading period. On the first view, this might seem contradictory compared to reports in the literature, where higher VA gains were observed at patients, receiving loading^13,28^. However, it is important to stress that the overall number of injections was kept constant between the scenarios with and without loading, presented in this work. This is a critical difference to performed comparisons in literature.

#### Impact of COVID-19

**Figure 6:**
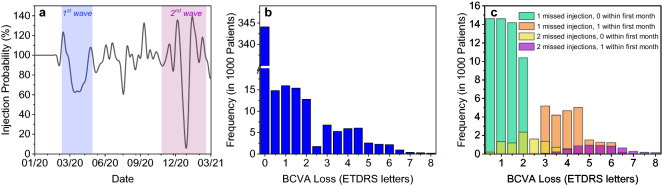
Impact of missed injections due to COVID-19 pandemic. (**a**) Percentage of performed injections based on daily Lucentis distribution numbers in Germany. (**b**) Histogram of BCVA loss due to COVID-19 from all patients, including patients without missed injections. (**c**) Histogram of BCVA loss due to missed injections, stratified according to number of missed injections and if one missed injection was within the first month of treatment. BCVA = best corrected visual acuity.

Figure 6a shows how the modelled frequency of performed injections decreased following the first and second COVID-19 waves. Injections decreased less steeply during the first wave than during the second, but the decrease lasted longer. The impact on VA outcome is shown in Fig. 6b. Mean BCVA loss across all 450,000 modelled patients was about 0.6 letters and was not regained later. Mean BCVA loss was relatively small because only about 25% of patients in this scenario had missed at least one injection. Considering only patients who missed at least one injection, mean loss was 2.2 letters.

Figure 6c shows the impact on VA independently for patients who missed 1 and 2 injections and the fact if one missed injection was within 30 days of treatment start.

About 73,000 patients missed one injection with zero missed in the first month after diagnosis (green bars), whereas 23,000 patients missed one injection with this being in the first month after diagnosis (orange bars). Mean BCVA losses related to those missed injections were 1.2 and 4.3 letters, respectively.

About 9000 patients missed two injections with no being missed in the first month after diagnosis (yellow bars), whereas 6000 patients missed two injections with one being in the first month after diagnosis (purple bars). Mean BCVA losses related to those missed injections were 2.5 and 5.7, respectively.

## Discussion and outlook

Many studies have shown that early treatment initiation, and frequent VEGF inhibitor injections during the maintenance phase results in better outcomes in patients with nAMD. We could confirm these observations using a mathematical prediction model based on real-world data. In addition, the model enriched those observation by a quantification, which is challenging to measure in a clean way, when considering a real world setting by nature and, hence, to date remained scarce.

The model analysis is based on real-world data of the NIS OCEAN, conducted between 2011 and 2016 in Germany. Mean VA gain in this real-world cohort laid in-between a ‘*natural course of disease*’ where the population was not-treated and a theoretical optimum scenario where treatment was simulated at an intensity observed under PRN schemes in clinical trials. Compared to the simulated optimum, mean VA in the real-world cohort increased less steeply during the first months of treatment and declined slowly after an initial peak.

An early start of treatment appeared to be decisive for the magnitude of the first improvement in VA. Initial mean VA gain was significantly larger in a scenario where treatment started without delay and a scenario where the delay was bound to a reasonable maximum of 2 weeks than in the real-world cohort. The difference between these scenarios did not diminish after the initial peak VA. Overall, 2 weeks delay until treatment start in the real-world cohort caused on average 2.5 letters loss compared to immediate treatment start. Four weeks delay or more caused on average 5 letters loss or more. The latter corresponds to a line and may be decisive for the ability of the patient to read.

Mean delay to treatment start was 20.3 days in the real-world cohort used in this study. For 29.1% of patients, the delay was longer than 4 weeks. Since the time of this study, awareness for the need to start treatment as early as possible has increased. In a newer real-world study conducted from 2015 to 2021, mean delay was only 8.5 days^29^. Also current guidelines recommend an interval of max. 14 days between first contact with a doctor due to symptoms and first injection^30^. Our simulation results confirm that these recent improvements saved vision for patients who would have waited longer in the past.

A correlation between delay to treatment start and VA outcome was established in several observational studies^16,17^. However, the analyses underline how important it can be for both studies and the clinical routine treatment to distinguish between the points in time (symptoms vs. initial diagnosis vs. first treatment) on the one hand, and to avoid any possible cause of delay on the other. Physiologically, the disease progresses during the delay, causing non-reversible damage to the retina and loss of vision. This may affect the potential for VA improvement in 2 ways: First, vision that was lost before treatment start may not be regained due to irreversible damage. Second, the improvement rate after onset of treatment may be slower as more VA is lost due to irreversible than due to reversible damage. In most previous correlation studies, only the second mechanism is captured, since the baseline for VA change was defined as treatment start, i.e., after the delay^17^. In the present study, only the first mechanism is considered, as baseline for VA change was defined before the delay but the improvement rate after treatment start was not varied in the model. Taking these results together, both mechanisms may be in place and the effect of delayed treatment start may even be larger than estimated in the present study.

After initial improvement in VA, regular monitoring and an adequately high frequency of injections are crucial to maintain or further improve the gained vision^5,14^. Many studies have shown that the frequency of injections and gained vision in real-world settings stayed below the potential that was demonstrated in clinical trials^11–13^. Our simulation results confirm this observation of relative undertreatment. In addition, they point to a new aspect: Accepting that the optimum of a complete loading period and sufficient frequency of maintenance injections may not be reached in real-world settings, the frequency of maintenance injections appears to be equally or even more important to preserve vision as a complete loading period. This was shown by a scenario with loading period and equally spaced injections, thereafter, compared to a scenario with equally spaced injections during the entire observation period (without loading period). The overall number of injections was equal in both scenarios; consequently, the injection frequency after the first 3 months was higher in the scenario without loading period. Simulations showed that after about 10 months of treatment, the scenario with higher injection frequency outweighed the benefit of a loading period.

More generally, the mathematical structure of the model implies that omitting or delaying an injection is more harmful when disease activity is high, and VA is low. This occurs before treatment start and in the situation of undertreatment during the maintenance phase. During the loading period, when disease activity could already be slowed down, delaying the treatment interval may be less detrimental.

Lastly, the model was successfully applied to estimate the impact of missed injections due to COVID-19 pandemic. The overall impact, averaged over the studied population, was predicted low with a mean loss of 0.8 letters. While the majority of patients in the simulated population was not affected, some patients lost 5 letters or more. Apparently, the impact of missed or delayed injections varies largely between individuals, depending on when the delay occurred (at which degree of disease activity), and how long and how often injections were omitted or delayed.

## Limitations

The model was developed to simulate mean VA outcome in a sufficiently large population under explicit treatment patterns. Accordingly, the model is not capable to predict individual patient outcomes. Reason for this limitation is that the individual variation between patients is captured only by the baseline parameters age, baseline VA and pre-treatment status and by the pattern of individual injections. Therefore, no conclusion can be made about individual patients who do not respond to the modelled treatment patterns as expected for the population mean.

Simulation results beyond a medium timescale (> ~ 18 months) are subject to large deviations. Reason is that long-term data are scarce compared to early data and model validity decreases with time. For this reason, only data until 18 months after start of treatment are considered in all scenarios. Long-term data of about 5 years would be needed to generate a robust model that is capable of long-term prediction.

The model is based on NIS data where documentation quality and observation artifacts may degrade the informative power of the data. In particular drop-outs may cause a bias due to potential elimination of worst disease courses.

The model was parametrized using solely data from patients, treated with ranibizumab. Thus, only ranibizumab treatment effects can be simulated with confidence using this model.

## Outlook

The validated VA prediction model is a valuable tool to predict population level VA response to individual treatment patterns in patients with nAMD treated with anti-VEGF. It provides the opportunity to systematically explore specified treatment patterns in real-world settings where there is usually no control group. Model scenarios may be of systematic nature or depict realistic situations such as the COVID-19 pandemic scenario. The good validation performance of the model provides confidence in simulation results when the model is applied within its purpose and limits. We hope that the model may become a widely used tool to improve treatment patterns for nAMD with reasonable effort.

## Supplementary Information

Supplementary Information.

## Data Availability

The datasets generated during the current study are available from the corresponding author on reasonable request.

## References

[1] Rosenfeld PJ (2006). Ranibizumab for neovascular age-related macular degeneration. N. Engl. J. Med..

[2] Brown DM (2006). Ranibizumab versus verteporfin for neovascular age-related macular degeneration. N. Engl. J. Med..

[3] Heier JS (2012). Intravitreal aflibercept (VEGF Trap-Eye) in wet age-related macular degeneration. Ophthalmology.

[4] Dugel PU (2020). HAWK and HARRIER: Phase 3, multicenter, randomized, double-masked trials of brolucizumab for neovascular age-related macular degeneration. Ophthalmology.

[5] Schmidt-Erfurth U (2014). Guidelines for the management of neovascular age-related macular degeneration by the European Society of Retina Specialists (EURETINA). Br. J. Ophthalmol..

[6] Khanna, S. *et al.* Current and upcoming anti-VEGF therapies and dosing strategies for the treatment of neovascular AMD: a comparative review. *BMJ Open Ophthalmol.* **4**, e000398 (2019). 10.1136/bmjophth-2019-000398 PMC6936465 31909196

[7] Gianniou C (2015). Two-year outcome of an observe-and-plan regimen for neovascular age-related macular degeneration: how to alleviate the clinical burden with maintained functional results. Eye.

[8] The CATT Research Group *et al.* Ranibizumab and bevacizumab for neovascular age-related macular degeneration. *N. Engl. J. Med.* **364**, 1897–1908 (2011). 10.1056/NEJMoa1102673 PMC3157322 21526923

[9] Ho, A. C. *et al.* Twenty-four-month efficacy and safety of 0.5 mg or 2.0 mg ranibizumab in patients with subfoveal neovascular age-related macular degeneration. *Ophthalmology* **121**, 2181–2192 (2014). 10.1016/j.ophtha.2014.05.009 25015215

[10] Silva R (2018). Treat-and-extend versus monthly regimen in neovascular age-related macular degeneration: Results with ranibizumab from the TREND study. Ophthalmology.

[11] Mehta H (2018). Real-world outcomes in patients with neovascular age-related macular degeneration treated with intravitreal vascular endothelial growth factor inhibitors. Prog. Retin. Eye Res..

[12] Ciulla TA, Hussain RM, Pollack JS, Williams DF (2020). Visual acuity outcomes and anti-vascular endothelial growth factor therapy intensity in neovascular age-related macular degeneration patients: A real-world analysis of 49 485 eyes. Ophthalmol. Retina.

[13] Holz FG (2020). Ranibizumab treatment in treatment-naive neovascular age-related macular degeneration. Retina Phila. Pa.

[14] Monés J (2020). Undertreatment of neovascular age-related macular degeneration after 10 years of anti-vascular endothelial growth factor therapy in the real world: The need for a change of mindset. Ophthalmologica.

[15] Okada M (2021). Defining nonadherence and nonpersistence to anti-vascular endothelial growth factor therapies in neovascular age-related macular degeneration. JAMA Ophthalmol..

[16] Sim, P. Y., Gajree, S., Dhillon, B. & Borooah, S. Investigation of time to first presentation and extrahospital factors in the treatment of neovascular age-related macular degeneration: A retrospective cross-sectional study. *BMJ Open* **7**, e017771 (2017). 10.1136/bmjopen-2017-017771 PMC5778287 29229653

[17] Lim JH (2012). Delay to treatment and visual outcomes in patients treated with anti-vascular endothelial growth factor for age-related macular degeneration. Am. J. Ophthalmol..

[18] Naravane, A. V. *et al.* Short term visual and structural outcomes of anti-vascular endothelial growth factor (anti-VEGF) treatment delay during the first COVID-19 wave: A pilot study. *PLOS ONE* **16**, e0247161 (2021). 10.1371/journal.pone.0247161 PMC7888661 33596257

[19] Sindal MD, Chhabra K, Khanna V (2021). Profile of patients receiving intravitreal anti-vascular endothelial growth factor injections during COVID-19-related lockdown. Indian J. Ophthalmol..

[20] Song W, Singh RP, Rachitskaya AV (2021). The effect of delay in care among patients requiring intravitreal injections. Ophthalmol. Retina.

[21] Holz FG (2010). The effects of a flexible visual acuity-driven ranibizumab treatment regimen in age-related macular degeneration: Outcomes of a drug and disease model. Invest. Ophthalmol. Vis. Sci..

[22] Mulyukov Z (2018). Neovascular age-related macular degeneration: A visual acuity model of natural disease progression and ranibizumab treatment effect. CPT Pharmacomet. Syst. Pharmacol..

[23] Agostini H (2020). Comparison of the efficacy of brolucizumab with natural disease progression in wet AMD using clinical data from the phase III HAWK and HARRIER trials and modelled placebo data. Curr. Eye Res..

[24] Wachtlin, J. *et al.* Use of imaging modalities in real life: impact on visual acuity outcomes of ranibizumab treatment for neovascular age-related macular degeneration in Germany. *J. Ophthalmol.* **2020**, e8024258 (2020). 10.1155/2020/8024258 PMC7382751 32724669

[25] Busbee, B. G. *et al.* Twelve-month efficacy and safety of 0.5 mg or 2.0 mg ranibizumab in patients with subfoveal neovascular age-related macular degeneration. *Ophthalmology* **120**, 1046–1056 (2013). 10.1016/j.ophtha.2012.10.014 23352196

[26] Prevalence of age-related macular degeneration in a large European cohort: Results from the population-based Gutenberg Health Study. 10.1007/s00417-014-2591-9. 10.1007/s00417-014-2591-9 24566902

[27] Lüdtke L, Jürgens C, Ittermann T, Völzke H, Tost F (2019). Age-related macular degeneration and associated risk factors in the population-based study of health in pomerania (SHIP-Trend). Med. Sci. Monit. Int. Med. J. Exp. Clin. Res..

[28] Sagkriotis, A. *et al.* Application of machine learning methods to bridge the gap between non-interventional studies and randomized controlled trials in ophthalmic patients with neovascular age-related macular degeneration. *Contemp. Clin. Trials* **104**, 106364 (2021). 10.1016/j.cct.2021.106364 33746023

[29] Khoramnia R (2021). Comparison of real-life treatment of naïve patients with ranibizumab over 24 months for neovascular AMD, diabetic macular edema, retinal vein occlusion and myopic CNV—results of the current interim analysis of the PACIFIC study. Invest. Ophthalmol. Vis. Sci..

[30] GERMAN SOCIETY OF OPHTHALMOLOGY. Stellungnahme der DOG, der RG und des BVA zur Therapie des diabetischen Makulaödems. *Ophthalmologe* **117**, 218–247 (2020).

