# Supplementary material for: A model to quantify the influence of treatment patterns and optimize outcomes in nAMD
Source: Sci Rep. 2022 Feb 18;12:2789. doi: 10.1038/s41598-022-06362-w (PMC8857272; doi:10.1038/s41598-022-06362-w)
Supplement: Supplementary file 1 — Supplementary Information. [file 41598_2022_6362_MOESM1_ESM.docx]

***A Model to Quantify the Influence of Treatment Patterns and Optimize Outcomes in nAMD***

*Focke Ziemssen, Hansjürgen Agostini, Nicolas Feltgen, Robert P. Finger, Christos Haritoglou,
Hans Hoerauf, Matthias Iwersen, Martina Porstner, Andreas Clemens, and Benjamin Gmeiner*

***Supplementary Information***

**Model**

Mulyukov *et al*. tested effects of age, sex and baseline VA on several model parameters. Only the effect of age on $E_{max,i}^{ss}$ (Equation 6) was significant and is kept in the model. However, baseline VA still has an implicit effect on VA due to the model structure given by Equation 4.

Mulyukov *et al*. applied a non-linear mixed model with fixed and random effects. Here, we estimated all parameters as fixed effects to keep the model parsimonious with the aim to predict population level mean VA change while prediction of individual level variation or estimation of p-values is not the focus of this study.

The model was parameterized by iterative least square estimation using Matlab R2020a on data of the OCEAN study. The parameters $\boldsymbol{g}_{\boldsymbol{ss}}$ **,**$k_{out}$ , $k_{drug}$ and $k_{E_{max}}$ were pre-specified (Mulyukov *et al*. 2018), all other parameters were estimated by the data. Missing VA values were not replaced. Simulations were run until Month 18 as data for later time points were scarce due to dropouts. Patients with missing baseline parameters were deleted listwise.

All parameter estimates are listed in S1.

**Supplementary tables and figures**

***Supplementary Table S1 | Parameter estimates of visual acuity prediction model, trained on data from the non-interventional study OCEAN.***

| Parameter | Unit | Description | Estimate |
| --- | --- | --- | --- |
| $\boldsymbol{g}_{\boldsymbol{ss}}$^a^ | letters | VA steady state under natural progression | 11 |
| $\boldsymbol{k}_{\boldsymbol{out}}$^a^ | 1/year | VA deterioration rate at natural progression | 0.19 |
| $\boldsymbol{k}_{\boldsymbol{drug}}$^a^ | 1/day | Rate constant for drug elimination from the vitreous | 0.077 (t_1/2_ = 9 days) |
| $\boldsymbol{E}_{\boldsymbol{max}}^{\boldsymbol{ss}}$ | - | Steady state maximum drug effect on VA at mean age | 6.1 |
| ${\boldsymbol{\Delta}\boldsymbol{E}}_{\boldsymbol{max}}^{\boldsymbol{0}}$ | - | Additional maximum drug effect on VA at baseline | 57.42 |
| $\boldsymbol{k}_{\boldsymbol{E}_{\boldsymbol{max}}}$^b^ | 1/day | Rate of E_max_ change | 0.046 (t_1/2_ = 15 days) |
| $\boldsymbol{EC}_{\boldsymbol{50}}$ | µg/L | Drug concentration for half of the maximum effect | 1.342 |
| $\boldsymbol{\alpha}$ | - | Scaling parameter for pre-treatment | 0.0281 |
| $\boldsymbol{\beta}$ | - | Age effect on $E_{max}^{ss}$ | -1.5 |
| ^a^ From literature; ^b^ Pre-specified. All other parameters were estimated by the OCEAN data.  VA = visual acuity | | | |

***Supplementary Figure S1 | Adjusted R² as a measure of model goodness of ft for mean BCVA gain predicted by the model vs. observed sample means in dependence of the number of patients in the sample. Samples were randomly drawn from data of the OCEAN study (3,631 patients) without replacement. Black and gray lines are mean and 95% prediction interval, respectively. BCVA = best corrected visual acuity***

***Supplementary Figure S2 | Mean BCVA change from baseline after 12 months of 0.5 mg monthly ranibizumab treatment as a function of patient’s baseline BCVA. Shaded area represents +/- one standard deviation. Age at baseline was drawn from normal distribution with mean (standard deviation) of 77.8 (8.2) years. BCVA = best corrected visual acuity***

***Supplementary Figure S3 | Mean BCVA change from baseline after 12 months of 0.5 mg monthly ranibizumab treatment as a function of patient’s age. Shaded area represents +/- one standard deviation. BCVA at baseline was drawn from normal distribution with mean (standard deviation) of 52.1 (21.3) ETDRS Letters. BCVA = best corrected visual acuity***

***Supplementary Figure S4 | Mean BCVA change from baseline in absence of treatment: MARINA RCT sham group with 95% confidence interval (blue) and model prediction with 95% prediction interval (red). BCVA = best corrected visual acuity***
